# IL-6 and diabetic kidney disease

**DOI:** 10.3389/fimmu.2024.1465625

**Published:** 2024-12-19

**Authors:** Lei Zhang, Futian Xu, Liyan Hou

**Affiliations:** ^1^ Pharmacy Department, Weihai Central Hospital Affiliated to Qingdao University, Weihai, China; ^2^ Logistics Management Department, Weihai Central Hospital Affiliated to Qingdao University, Weihai, China

**Keywords:** IL-6, inflammation, diabetic kidney disease, IL-6 classical signaling, IL-6 trans-signaling pathway

## Abstract

Diabetic kidney disease (DKD) is a severe microvascular complication of diabetes associated with high mortality and disability rates. Inflammation has emerged as a key pathological mechanism in DKD, prompting interest in novel therapeutic approaches targeting inflammatory pathways. Interleukin-6 (IL-6), a well-established inflammatory cytokine known for mediating various inflammatory responses, has attracted great attention in the DKD field. Although multiple *in vivo* and *in vitro* studies highlight the potential of targeting IL-6 in DKD treatment, its exact roles in the disease remains unclear. This review presents the roles of IL-6 in the pathogenesis of DKD, including immunoinflammation, metabolism, hemodynamics, and ferroptosis. In addition, we summarize the current status of IL-6 inhibitors in DKD-related clinical trials and discuss the potential of targeting IL-6 for treating DKD in the clinic.

## 1 Introduction

Diabetic kidney disease (DKD) is the leading cause of end-stage kidney disease (ESKD) worldwide and is the major contributor to diabetes mortality rate. Despite recent progress in the development of new drugs for DKD, the residual risk still remains (1). Identifying potential novel targets and therapeutic drugs for DKD is of great clinical significance. Numerous studies have highlighted inflammation as a crucial factor in the occurrence and progression of DKD (2, 3). DKD now is also acknowledged as an inflammatory disorder (4). Rayego-Mateos et al. propose prioritizing anti-inflammatory interventions in the management of DKD by 2030 (5).

Several important reviews highlight the critical role of inflammatory factors, especially interleukin-6 (IL-6), in the development of type 2 diabetes mellitus (T2DM) and its associated kidney diseases (2, 6). Research has demonstrated significantly elevated IL-6 levels in T2DM patients, which closely correlate with declining renal function (7). Several large cohort studies have confirmed the direct relationship between high circulating IL-6 levels and reduced kidney function in patients with chronic kidney disease (CKD) (8, 9). Compared to other cytokines such as interleukin-1 (IL-1) and tumor necrosis factor (TNF), anti-IL-6 therapy is more effective in improving serum lipoprotein levels in patients with rheumatoid arthritis (RA) (10). Additionally, IL-6 is more relevant to the 5-year all-cause and cardiovascular mortality risks in patients with CKD and ESKD than TNF and fibrosis markers (11). Among patients with ESKD, plasma IL-6 levels are more effective in predicting mortality risk than IL-1, interleukin-18 (IL-18), and TNF (12). Based on these findings, therapeutic strategies targeting IL-6 show significant potential in the management of DKD. For instance, studies using the IL-6 receptor blocker tocilizumab (TCZ) in mouse models have demonstrated its ability to ameliorate the pathological changes associated with DKD (13), further supporting the feasibility of targeting IL-6. The fact that TCZ has been successfully utilized in clinical settings for treating inflammatory diseases such as RA suggests that targeting IL-6 could represent a promising strategy for DKD. However, the exact physiological and pathological roles of IL-6 in DKD remain unresolved. In this article, we summarize the current understanding of IL-6 signaling in DKD to facilitate in-depth studies of potential therapeutic strategies for targeting IL-6 in the treatment of DKD in the future.

## 2 IL-6 and its signaling pathways

IL-6 plays a key role in multiple biological processes, including immune responses, vascular diseases, developmental processes, metabolic regulation. This pleiotropic activity has made it a focal point of research, particularly in inflammatory pathological states. When exploring the mechanisms of IL-6, it is important to understand its receptor system. The IL-6 receptor system includes both membrane-bound interleukin-6 receptor alpha chain (also known as mIL-6R) and soluble forms of IL-6Rα (also known as sIL-6R), with glycoprotein 130 (gp130, the IL-6Rβ subunit) playing a central role in signaling. IL-6Rα binds to IL-6, while gp130 is crucial for transmitting the signal inside the cell.

**Figure 1:**
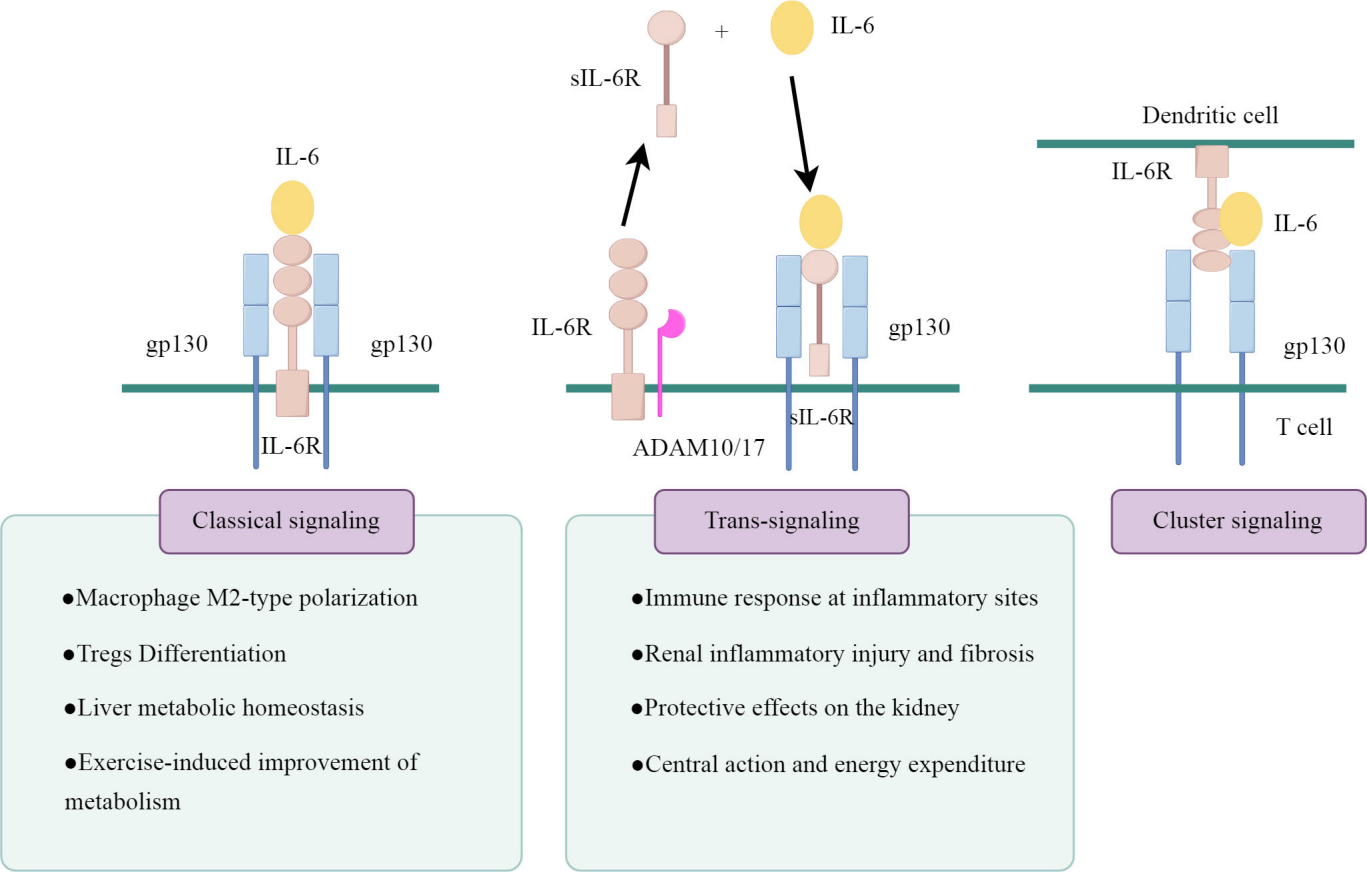
The signaling patterns of IL-6.

IL-6 actions are mediated by three distinct intracellular signaling pathways, as illustrated in **Figure 1** (drawn by Figdraw). The first signaling pathway is the classical signaling pathway mediated through mIL-6R. IL-6 binds to mIL-6R and induces the homodimerization of the gp130 receptor chain, which recruits Janus kinases (JAKs) that activate each other and trigger the downstream signaling pathways, including the SHP-2/ERK MAPK pathway and the JAK/STAT pathway (14). This classical signaling pathway of IL-6 mainly occurs in a few types of cells, primarily found in immune cells (neutrophils, macrophages and CD4^+^ T cells) and resident cells (hepatocytes, pancreatic cells and podocytes) (15–17).

The second is the sIL-6R-mediated signaling system, known as IL-6 trans-signaling. sIL-6R is produced either by the proteolytic shedding of mIL-6R mediated by metalloenzymes such as a disintegrin and metalloprotease 10 (ADAM10) and a disintegrin and metalloprotease 17 (ADAM17), or by the selective splicing of IL-6R mRNA (18). sIL-6R binds to IL-6 to form a complex, which further activates gp130 and transmits the signal. gp130 is a signal transducer shared by all IL-6 family members, and theoretically, the IL-6/sIL-6R complex is capable of activating all cells in the body because of the widespread expression of gp130. However, this trans-signaling is highly regulated by naturally occurring soluble gp130 (sgp130), which binds to the IL-6/sIL-6R complex with high affinity and specifically blocks the IL-6 trans-signaling pathway, thus constituting a physiological buffer for the IL-6 trans-pathway (19).

In cells lacking mIL-6R, the trans-signaling pathway of IL-6 is their only signal transduction pathway. However, in cells expressing mIL-6R, the situation becomes more complex. Typically, these cells undergo parallel activation of both the classical and trans-signaling pathways. sIL-6R can competitively bind to IL-6 with mIL-6R, and the ratio of cell surface IL-6Rα/gp130 expression determines the strength of trans-signaling versus classical signal transduction (20). Moreover, when the molar concentration of sIL-6R exceeds that of IL-6, sgp130 can inhibit classical signal transduction (21).

The third pathway is cluster signaling pathway, primarily occurring between dendritic cells (DCs) and T cells in experimental mice (22). IL-6 binds to IL-6Rα on the surface of DCs, forming a complex that subsequently interacts with gp130 on the T cell membrane. This interaction induces gp130 dimerization, activating IL-6 signaling and initiating the Th17 response. However, its role in humans remains unconfirmed.

In summary, the IL-6 signaling system transmits information between cells through different receptor forms and regulatory mechanisms, thereby influencing disease progression. In recent years, researchers have also explored the pleiotropic roles of IL-6 at molecular genetic level. For instance, an IL-6 meta-analysis of genome-wide association studies (GWAS) identifies potential IL-6-modulating genes, including the interleukin-1 receptor antagonist gene (IL1RN), human leukocyte antigen (HLA), and IL-6R, which have roles in immunity and inflammation (23). Another genetic study links the IL6 single nucleotide polymorphism (SNP), inflammation and its association with ESKD (24). Future research on these novel aspects of IL-6 at the genetic level may help broaden the understanding of new potential signaling pathways, thereby elucidating the complex physiological functions of IL-6. In this context, the exploration of the relationship between IL-6 and DKD is of particular importance, as IL-6 plays a pivotal role in diabetes-related inflammatory responses and may significantly affect kidney structure and function.

## 3 The relationship between IL-6 and DKD

First, genetic studies focusing on immune responses, as well as systematic reviews and meta-analyses of GWAS, have revealed that IL-6 gene variants are associated with an increased risk of DKD (25–27). Second, IL-6 is associated with kidney injury in DKD. IL-6 expression is significantly upregulated in the kidneys of rat models with DKD (28, 29), and their kidney weight and proteinuria levels are correlated with renal IL-6 levels (28). Several human studies have indicated a marked increase in serum IL-6 levels among individuals with DKD (30–32). Furthermore, there exists a direct correlation between high glycated hemoglobin A1c (HbA1c) levels (33) and early glomerular structural abnormalities (31) in patients with T2DM in relation to serum IL-6 levels. Moreover, circulating IL-6 levels in patients with DKD are elevated compared to those in diabetic individuals without kidney disease (30, 32). IL-6 expression is found to be increased in the kidneys of patients with CKD or DKD (34, 35), particularly in areas of mesangial expansion, tubular infiltration and atrophy (35). Additionally, infiltrating immune cells in the damaged kidneys, including macrophages, T cells, and basophils, exhibit elevated levels of IL-6 expression (36–38), which further drives the progression of the inflammatory response.

More importantly, IL-6 emerges as a promising biomarker for predicting the progression of DKD. Clinical studies have shown that plasma IL-6 is an independent predictor of mortality in patients with advanced CKD (39), and plasma IL-6 levels in CKD patients increase with the CKD stage. Elevated plasma IL-6 levels in patients with T2DM are associated with deterioration in kidney function, independent of baseline kidney function or proteinuria (7). Several large cohort studies have confirmed a direct correlation between IL-6 levels and kidney function, with high IL-6 levels significantly associated with decreased kidney function in individuals with or without CKD (8, 9).

The abnormal expression of IL-6 in animal models and human studies of T2DM and DKD reveals its close relationship with these diseases, highlighting the necessity of focusing on the key roles of the inflammatory cytokine IL-6. Therefore, the potential mechanisms by which IL-6 affects DKD are worthy of further investigation.

## 4 Potential mechanisms of IL-6 Involvement in the regulation of DKD

The regulatory mechanisms of IL-6 in DKD are complex. Here, we explore the effects of IL-6 in relation to immunoinflammation, kidney cell function, glucolipid metabolism, renin-angiotensin-aldosterone system (RAAS) activation, and iron homeostasis, aiming to comprehensively elucidate the central role of IL-6 in the development of DKD.

### 4.1 Inflammatory immune cells: IL-6 regulates the initial and resolution phases of inflammation

From a pathological perspective, inflammation in DKD begins with an immune response initiated by the immune system, which is essential for the clearance of infectious factors. Once the threat signals have been eliminated, it is vital to restrain this response to prevent excessive tissue damage and reduce chronic inflammation (40). CD45^+^ immune cells play an important role in this process, so it is crucial to study the regulatory mechanisms of IL-6 on renal CD45^+^ immune cells.

In normal kidneys, a small number of CD45^+^ immune cells are detected; however, in the kidneys of DKD patients, these cells increase in the early stages (41) and may decrease in the late stages, leading to a dampened immune response and exacerbated tissue damage (42). The types of infiltrating immune cells vary across different renal regions: in diabetic glomeruli, macrophages predominate (43), whereas in crescentic glomerulonephritis (CGN), monocytes are prevalent within the glomeruli, and T cells are primarily found in the surrounding areas (41, 44). Monocytes (especially macrophages) and T cells play pivotal roles in renal injury and fibrosis (41, 44–46). These infiltrating immune cells, along with kidney cells, release various cytokines, including IL-6, which regulates immune cell activation and proliferation, modulates local inflammation, and participates in tissue repair.

Key events in the transition to the resolution phase include the apoptosis and clearance of infiltrating neutrophils, the activation of anti-inflammatory M2-type macrophages, and the differentiation of regulatory T cells (Tregs) (40). Interestingly, IL-6 plays an important role in both the initiation and resolution phases of inflammation. In the initiation of inflammation, neutrophils are the first to accumulate in tissues, followed by the release of sIL-6R, which initiates trans-signaling in the intrinsic renal cells, promoting the expression of inflammatory chemokines and intercellular adhesion molecules (47). IL-6 transactivation plays an important role in the regulation of neutrophil transport and clearance (48), driving the transition of neutrophil recruitment to monocyte recruitment, and inducing the shift from innate immunity to adaptive immunity (49).

Enhanced renal macrophage infiltration is evident throughout all stages of DKD. Macrophages exhibit two activation phenotypes: pro-inflammatory M1 and the anti-inflammatory M2. It has been demonstrated that M1 and M2 macrophages primarily infiltrate the glomeruli and interstitium of diabetic kidneys (41). In the early stages of DKD, M1-type macrophages are first recruited and then proliferate in the kidneys (50). Subsequently, IL-6 activates the IL-6Rα on the surface of M1 macrophages, inhibiting M1 proliferation while promoting the polarization of M2 macrophages. This process alleviates renal inflammation and facilitates tissue repair (51). The classical IL-6 signaling pathway may a potential therapeutic strategy to improve DKD, as its activation enhances the repair of inflammatory renal injuries (51, 52). However, in the late stages of DKD, M2 macrophages may transform into fibroblasts, contributing to fibrosis in chronic kidney disease, including DKD (53, 54). Thus, a deeper investigation into the phenotypic changes of macrophages and their roles at various stages of DKD is essential for optimizing treatment strategies.

DCs are essential antigen-presenting cells in the adaptive immune response, with IL-6 playing multiple roles in their regulation. IL-6 inhibits DC development (55) and maintains them in an immature state (56), thereby limiting their capacity for antigen presentation and T cell activation. However, under certain conditions, IL-6 can promote DC maturation and further enhance T cell responses (57). Additionally, IL-6 secreted by DCs induces the production of Th17 cells, promoting adaptive immune responses (58, 59).

T cells play a crucial role in the progression of DKD, particularly CD4^+^ T helper cells (Th). Effector CD4^+^ T helper cells can be classified into four primary subsets: Th1, Th2, Th17, and Treg. Studies have shown that the decreased Treg/Th17 ratio is an essential facilitator of worsening kidney function in diabetic patients (60, 61). IL-6 plays a significant role in regulating the Th17/Treg balance. Early studies suggest that IL-6 induces pathogenic Th17 responses in synergy with TGF-β while also suppressing Treg differentiation, ultimately resulting in tissue inflammation and damage (62). However, recent research indicates the duality of IL-6 action *in vivo*. The expression of mIL-6R is predominantly found in peripheral naive CD4^+^ or central memory T cells, whereas at inflammatory sites, IL-6 trans-signaling becomes essential for activated and memory T cells due to the downregulation of mIL-6R expression (63). At the initiation stage of inflammation, activation of the classical IL-6 pathway in naive CD4^+^ T cells leads to the development and functional maintenance of Th17 cells (64). At inflammatory sites, T cells rely on IL-6 trans-signaling to facilitate Th17 differentiation and recruitment (63, 65, 66) while simultaneously inhibiting apoptosis, which leads to persistent infiltration (67, 68). In the resolution phase of inflammation, the classical activation of IL-6 promotes Treg differentiation and their trafficking to the kidneys, aiding the repair of damaged kidneys (69). Several studies have emphasized that interference with the classic IL-6 pathway reduces M2 macrophages and enhances Th17 responses, worsening renal injury (51, 52). Further research has indicated that M2 macrophages regulate adaptive immune responses through various mechanisms, including promoting Treg differentiation, enhancing their ability to suppress Th17 cells, and facilitating the migration of Tregs to sites of renal inflammation (70–72), which indirectly inhibits Th17 cell activity. Based on these findings, selective interference with the IL-6 trans-signaling may exert better therapeutic effects on chronic inflammatory injury, but effects on initial immune responses must be carefully considered.

Global inhibition of IL-6 signaling enhances pro-inflammatory Th1 and Th17 responses in mouse kidneys, exacerbating tissue and functional damage (51). This is consistent with the dual mechanisms of IL-6, indicating that IL-6 may have a protective role under certain circumstances. In CGN models, elevated circulating sIL-6R levels are closely associated with the aggravation of renal injury (52). During advanced disease, inhibitors of the IL-6 pathway, especially those targeting IL-6 trans-signaling, significantly inhibit the recruitment of M2 macrophages, CD4^+^ T cells in mouse kidneys (73, 74), thereby alleviating renal fibrosis. These studies confirm the effectiveness of selective inhibition of IL-6 trans-signaling in controlling kidney inflammation.

IL-6 plays a complex and multifaceted role in the inflammatory process of DKD, participating in both the initiation and resolution of inflammation. In these processes, IL-6 exerts both pro-inflammatory and anti-inflammatory effects, with its specific actions related to the signaling patterns involved. Further studies should be conducted to elucidate the specific mechanisms of IL-6 in various types of cells and stages to develop more precise and effective therapeutic strategies. Moreover, attention should also be paid to the potential impacts of the dual roles of IL-6 for effective inflammation control.

### 4.2 Effects of IL-6 on renal cells: good or bad?

**Figure 2:**
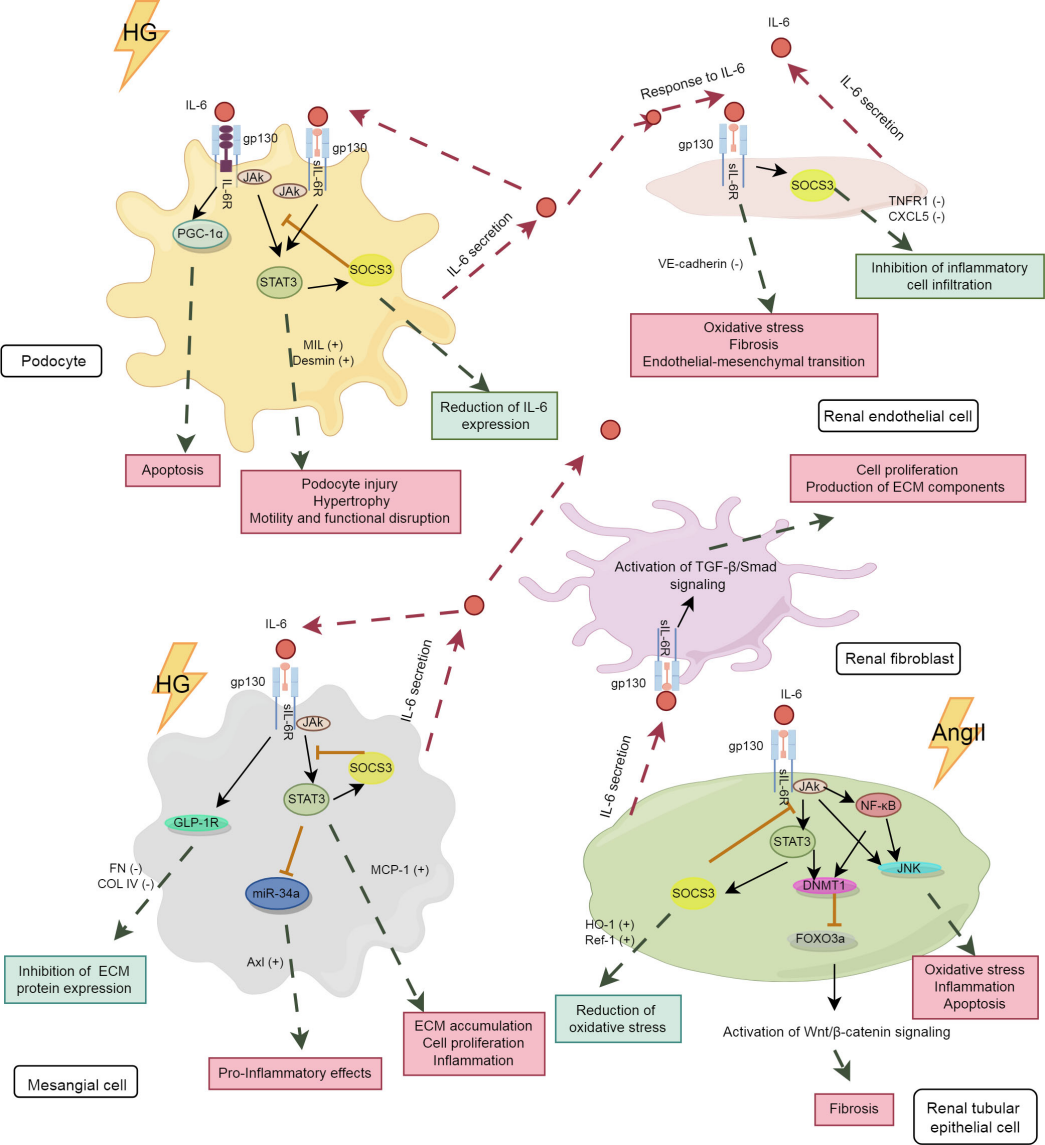
The IL-6 signaling pathway in kidney cells. The pink boxes in the panel denote the damaging effects, whereas the blue-green boxes represent the protective effects. HG, high glucose; PGC-1α, peroxisome proliferator-activated receptor gamma coactivator 1 alpha; TNFR1, tumor necrosis factor receptor 1; CXCL5, C-X-C motif chemokine ligand 5; GLP-1R,glucagon-like peptide-1 receptor; miR-34, microRNA-34; AngII, angiotensin II; MCP-1, monocyte chemoattractant protein-1; DNMT1, DNA methyltransferase 1; FOXO3a, forkhead box O 3a; HO-1, heme oxygenase-1; Ref-1, redox effector factor 1; VE-cadherin,vascular endothelial-cadherin; MLC, myosin light chain; COL IV, collagen type IV.

The kidney consists of renal parenchyma and interstitium, with the renal parenchyma serving as the primary functional component, encompassing the glomeruli, renal tubules, and renal vasculature. The expression levels of IL-6R mRNA and protein in the renal parenchyma are very low, thus impeding the transduction of IL-6 classical signaling in the kidneys (75). However, podocytes have been demonstrated to express membrane-bound IL-6R, allowing for the transduction of both classical and trans-signaling pathways of IL-6, while other renal cells predominantly transduce IL-6 trans-signaling (76). The analysis of differences in the effects of IL-6 signaling pathways among key kidney cell types, including podocytes, renal endothelial cells (RECs), mesangial cells (MCs), renal tubular epithelial cells (RTECs), and renal fibroblasts, will enhance the understanding of the “good or bad” effects of IL-6 on these cells, thereby uncovering the intrinsic relationship between IL-6 and DKD. The IL-6 signaling pathway in various kidney cells is depicted in **Figure 2**.

#### 4.2.1 Podocytes and RECs

Podocytes and RECs are essential components of the glomerular filtration barrier. IL-6 plays a significant role in regulating the structure and function of both podocytes and RECs. As shown in **Table 1**, under diabetic conditions, high glucose induced podocytes to secrete IL-6, which activated the IL-6/gp130/PGC-1α and IL-6/gp130/JAK2/STAT3 signaling pathways, potentially leading to podocyte apoptosis (77), hypertrophy (78), and cytoskeletal disruption (79). Baseline IL-6 may exert a protective effect on podocytes, as IL-6-deficient mice develop acute kidney injury (AKI) with podocyte dysfunction (80). Under certain conditions, IL-6 secreted by podocytes promoted the expression of suppressor of cytokine signaling 3 (SOCS3) in RECs, thereby inhibiting IL-6 signaling and reducing neutrophil recruitment (81). However, these effects were not observed in co-cultures with human umbilical vein endothelial cells. This indicates the tissue-specific actions of IL-6. As the only renal cells expressing IL-6R, podocytes are studied to explore their specific signaling patterns. It has been shown that both classical and trans-signaling pathways of IL-6 are harmful to podocytes under high glucose conditions (76). More studies are needed to further elucidate the underlying mechanisms involved.

**Table 1:** Effects of IL-6 on kidney cells.

Cell type	Role of IL-6	Refs
Podocyte	Promoted proliferation, apoptosis, and growth arrest, stimulation of hypertrophy, induction of excessive motility and functional disruption, protection of podocytes in AKI models	(76–79, 175)
Endothelial cell	Induction of inflammatory phenotype, induction of endothelial-mesenchymal transition and fibrosis, increased permeability, inhibited neutrophil recruitment to inflamed cells	(81, 83–85)
Mesangial cell	Promotion of proliferation, enhanced chemokine expression, induction of apoptosis and fibrosis, reduced abundance of fibronectin and type IV collagen	(86, 88–92, 176)
Tubular epithelial cell	Induction of apoptosis, mesenchymal transition and fibrosis, promotion of immune cell accumulation, attenuated oxidative stress damage	(75, 93–96, 177)
Renal fibroblast	Increased ECM production, activation of the transition to myofibroblasts	(97, 98, 160)

AKI, acute kidney injury; ECM, extracellular matri.

In addition, high glucose stimulates the secretion of IL-6 by RECs (82), inducing an inflammatory phenotype in RECs with increased levels of oxidative stress and fibrosis markers (83). Long-time exposure to IL-6 results in the loss of typical markers of RECs, contributing to renal fibrosis (84). In immunoglobulin A nephropathy, IL-6 suppressed the expression of vascular endothelial cadherin through trans-signaling pathway, causing increased permeability of RECs (85).

#### 4.2.2 MCs

The persistent activation of the IL-6 pathway in MCs is an important mechanism of the early glomerulopathy in DKD. As an autocrine signal, IL-6 induces the expression of chemokines such as monocyte chemoattractant protein-1 (MCP-1) in MCs and influences immune cells through paracrine effects, thereby exacerbating immune-inflammatory damage in the glomeruli (86). Furthermore, studies have shown that the activation of Axl receptor tyrosine kinase (Axl) is closely associated with early glomerular hypertrophy in DKD (87), with the IL6/STAT3/miRNA-34a pathway playing a significant role in this process (88). In a hyperglycemic environment, the elevation of IL-6 in MCs led to cell apoptosis (89) and promoted cell proliferation, inflammation and fibrotic changes through the IL-6/JAK2/STAT3 pathway, thereby hastening the progression of DKD (90).

IL-6 trans-signaling plays an important role in regulating the inflammatory response of MCs. Its activation markedly induced MCP-1 synthesis in MCs, leading to glomerular inflammation (91). However, IL-6 may exhibit tissue-specific anti-inflammatory and pro-inflammatory effects under different conditions. For example, in RTECs, IL-6 induced increased expression of extracellular matrix (ECM) proteins, whereas it inhibited ECM protein expression in MCs (92). The pro-inflammatory fibrotic effect observed in RTECs cannot be simply defined as detrimental, as initial fibrosis can promote wound healing. More studies are needed to clarify the physiological significance of the differential effects of the IL-6 trans-signaling pathway in various tissues of the same organ.

#### 4.2.3 RTECs and renal fibroblasts

Activation of renal fibroblasts and phenotypic conversion of RTECs are key factors in tubulointerstitial fibrosis (TIF). The activation of the IL-6 trans-pathway is closely associated with TIF. High glucose activated the IL-6/JAK2/STAT3 signaling pathway in RTECs, leading to inflammatory damage and cell apoptosis (93). Increased IL-6 induced epithelial-mesenchymal transition of RTECs (94) through the downstream STAT3 and DNMT1/FOXO3a/Wnt/β-catenin axis, promoting renal fibrotic lesions in AKI (95, 96). Furthermore, IL-6 released by RTECs promotes the proliferation of adjacent fibroblasts and the excessive production of fibrotic matrix (97, 98), accelerating renal fibrosis progression. Although IL-6 promotes renal injury in AKI (95, 99, 100), its trans-signaling also limits oxidative stress and promotes kidney repair (75), underscoring the dual role of IL-6 in tissue injury. This dual role may protect local tissues and organs from damage caused by acute inflammatory stimuli.

Animal studies further confirm the role of IL-6 in renal fibrosis and diabetic kidney injury. IL-6 knockdown markedly reduces renal fibrosis in type 1 diabetic mice (101). In type 2 diabetic nephropathy models, IL-6 neutralization inhibits nod-like receptor family pyrin domain-containing 3 (NLRP3) inflammasome and downstream JAK2/STAT3 signaling, thereby alleviating oxidative stress and fibrotic injury (13, 90). Additionally, IL-6 elevates serum fibroblast growth factor 23 (FGF23) levels via sIL-6R (99), promoting the development of acute and chronic renal failure. In models of unilateral ureteral obstruction and ischemia reperfusion, specific blockade of IL-6 trans-signaling attenuates inflammation and fibrosis in renal tissues (74, 102). Consequently, the up-regulation of IL-6 trans-signal is closely associated with the progression of DKD.

### 4.3 Complexity of IL-6 regulation of glycolipid metabolism

Although IL-6 theoretically has the potential to regulate metabolism, adverse effects such as dyslipidemia have been observed with IL-6 inhibitors (e.g., TCZ) in clinical trials (103). This prompts us to revisit the complex roles of IL-6 in metabolic regulation. Therefore, we review the literature related to glycolipid metabolism, focusing on energy metabolism, T2DM models, and clinical trials, to explore the specific mechanisms by which IL-6 regulates glycolipid metabolism.

#### 4.3.1 IL-6 regulates metabolism in a tissue-specific manner

IL-6 exhibits inconsistent effects among different peripheral target tissues of insulin, primarily including the liver, adipose tissue, and muscle, indicating the tissue-specific effects of IL-6 on metabolic regulation. The IL-6R on hepatocytes allows the intracellular classical signaling pathway that is essential for preventing metabolic changes and inflammation (104). Administration of IL-6-neutralizing antibodies to mice aggravates methionine-choline-deficient diet-induced hepatic steatosis (105), while hepatocyte-specific IL-6Rα deficiency leads to liver inflammation and reduced insulin sensitivity (106). In chronic liver disease, the downregulation of mIL-6R expression in hepatocytes favors the transduction of the IL-6 trans-signaling pathway, ultimately resulting in liver cirrhosis (107, 108). Increased plasma concentrations of IL-6 trans-signaling components show a clear correlation with HbA1c levels in patients with diabetes concomitant chronic liver disease (107). These studies suggest that the classical pathway mediated by hepatic IL-6R may maintain metabolic homeostasis in the early stages of the disease, but as the disease progresses, IL-6 trans-signaling predominates, leading to metabolic disturbances and inflammation.

IL-6 is strongly associated with lipid metabolic changes in both obese individuals and those with T2DM (109, 110), and adipose tissue is the primary source of IL-6 *in vivo*. Adipocytes predominantly transmit IL-6 trans-signaling (111), although there are also studies indicating the presence of IL-6R on adipocytes and suggesting its role in mediating liraglutide-induced hypoglycemic effects (112). Acute increases in IL-6 in adipose tissue have been shown to improve metabolism and blood glucose levels in obese mice (113), whereas prolonged elevations of IL-6 may contribute to increased lipolysis and leptin production, as well as insulin resistance (114). Moreover, IL-6/sIL-6R signaling pathways activated by high-fat diets have been found to exacerbate inflammation in adipose tissue of mice (115). Inhibition of IL-6 signaling in adipose tissue has been associated with reduced SOCS3 expression in hepatocytes and improved insulin sensitivity in mice. Selective inhibition of IL-6 in adipose tissue decreased SOCS3 expression in hepatocytes, thereby improving insulin sensitivity in mice (116). Thus, the chronic elevation of IL-6 in adipose tissue of obese patients is linked to metabolic deterioration.

Recent studies have indicated a connection between the classical IL-6 pathway and the metabolic benefits brought about by exercise. During exercise, IL-6, released by muscle tissue, stimulates the uptake and breakdown of glucose and fatty acids in muscle fibers through an osteocalcin-dependent mechanism by activating the IL-6 classical pathway in osteoblasts to optimize motor function (117). Moreover, IL-6 produced by muscles acts through IL-6R to support the retention and functional enhancement of Tregs in muscles, thereby improving muscle mass (118).

In addition to peripheral tissues (liver, adipose, and muscle), IL-6 is also involved in central metabolic regulation. Several studies have confirmed that central IL-6 activation can prevent high-fat-diet-induced obesity and insulin resistance in mice (119–121). Central IL-6 is closely related to body energy expenditure (120, 122). IL-6 trans-signaling plays a dominant role in its central effects. The trans-signaling of IL-6 in the paraventricular nucleus of the hypothalamus is essential for inhibiting feeding behavior and enhancing glucose homeostasis in mice (121).

#### 4.3.2 IL-6 in models of T2DM

Patients with T2DM or DKD are usually accompanied by elevated circulating IL-6 levels. This chronic elevation of IL-6 similar to those observed in chronic inflammation, tends to disrupt the organismal metabolic homeostasis. TCZ treatment notably improved circulating glucagon, glucose and HbA1C levels in rhesus monkeys with spontaneous obesity or T2DM (123).

In contrast to chronic IL-6 actions, it is interesting to note that acute IL-6 infusions (at physiological concentrations) may improve the metabolism of patients with T2DM. In experimental designs, acute or intermittent IL-6 injections are often used to mimic the acute elevation of IL-6 post-exercise. Acutely elevated IL-6 has been shown to improve glucose tolerance in rodents and postprandial glucose in healthy volunteers (113, 124). In T2DM patients, acute infusions of IL-6 (up to the concentration after acute exercise), while not altering glucose turnover or postprandial glucose in patients, both notably reduced insulin levels in blood, implying an enhancement of insulin sensitivity (110, 124). This insulin-sparing effect may help to retain the function of pancreatic β-cells in diabetic patients. A single IL-6 injection improved insulin sensitivity in obese and type 2 diabetic mice, but failed to increase insulin secretion after pancreatic β-cell destruction in mice (113). It appears that the improvement of insulin sensitivity by IL-6 in T2DM patients is dependent on the preservation of pancreatic β-cell function. Furthermore, acute infusions of IL-6 can enhance fatty acid metabolism and systemic energy metabolism in elderly participants with T2DM (125), which emphasizes the lipolytic effects of IL-6 and may contribute to weight loss in them.

The pathological microenvironment induces changes in cell phenotypes, and the beneficial effects of IL-6 on metabolism observed in healthy volunteers cannot be simply extrapolated to diabetic patients. In healthy volunteers, acute IL-6 infusions delay gastric emptying and lower postprandial glucose levels. However, in patients with T2DM, IL-6 infusions delay gastric emptying but do not considerably reduce postprandial blood glucose levels (124). The underlying mechanisms of these differences remain unknown. It has been clearly demonstrated that individuals with T2DM show reduced responsiveness of myotubes to IL-6. While skeletal muscle cells from healthy volunteers can increase glucose uptake in response to IL-6 stimulation, myotubes from individuals with T2DM only respond to insulin *in vitro*, not to IL-6 (126).

Activation of the classical pathway of IL-6 may be important for improving metabolic disorders in patients with T2DM. Individuals with elevated serum levels of sIL-6R and/or sgp130 are at an increased risk of metabolic syndrome (127, 128). Prolonged exercise training can reduce plasma sIL-6R levels (129), thereby shifting the IL-6 signaling axis toward the classical IL-6 signaling mode. In addition, the researchers construct a novel compound, IC7Fc, which preserves the metabolic benefits of IL-6R. This compound improves hyperglycemia and glucose tolerance levels, while also reducing body weight and liver cirrhosis as well as promoting muscle and bone mass (130). Although blocking the trans-signaling pathway of IL-6 improves ATM accumulation induced by a high-fat diet, it does not alleviate systemic insulin resistance (131). Therefore, modulation of the IL-6 signaling pathway toward the classical model may be more effective in correcting metabolic disorders than simply blocking the trans-signaling pathway.

In the development of IL-6 agents, attention should also be paid to the potential impact of IL-6 blockade on the beneficial outcomes of exercise. A small-scale, double-blind clinical trial demonstrated that IL-6 blockade led to decreases in acute post-exercise and postprandial active GLP-1 concentrations among obese and type 2 diabetic individuals, albeit with a transient rather than sustained effect (132). Apart from that, another study showed that TCZ abolished exercise-induced reduction in visceral fat and increases circulating cholesterol levels in obese subjects (133).

#### 4.3.3 Metabolic effects of IL-6 inhibitors observed in clinical trials

RA and diabetes share common pathogenic mechanisms, which involve chronic inflammation, autoimmunity, and insulin resistance and beyond. In recent years, research on RA has been shifting focus towards its complications of diabetes. Currently, the number of clinical trials investigating IL-6 inhibitors for diabetes treatment is limited. Therefore, large-scale clinical trials of TCZ in RA patients aid in the assessment of the metabolic effects of IL-6.

As shown in **Table 2**, beneficial effects on glucose metabolism and muscle mass are observed with TCZ therapy. In non-diabetic RA individuals, TCZ treatment notably improves insulin sensitivity and enhances muscle mass (134–136). In RA patients with T2DM, TCZ demonstrates a notable reduction in HbA1c levels and a decrease in daily prednisolone doses (137). Despite a case report also exists where RA patients developed type 1 diabetes after 17 months of TCZ treatment (137), the precise underlying mechanism remains unclear. Considering the genetic background of strong susceptibility genes in the patient, it is also possible that TCZ may delay the onset of type 1 diabetes.

**Table 2:** Metabolic effects of IL-6 inhibitors observed in RA clinical trials.

Year	Diseases/Patients (n)	Interventions	Major results	Refs
2004	RA (n=164)	MRA	Elevation in TC, LDL-C, and triglyceride levels, mild liver disease, transient decrease in white blood cell counts	(138)
2011	RA with T2DM (n=10) RA without T2DM (n=29)	TCZ	T2DM patients: significant reduction in HbA1c Non-T2DM patients: slight decrease in HbA1c, no hypoglycemia observed	(137)
2011	RA (n=22)	TCZ	Elevated TC and HDL levels, decreased arterial stiffness	(140)
2013	RA with T2DM (n=50)	TCZ	Decrease in insulin/glucose ratio, improved insulin sensitivity	(134)
2015	RA (n=132)	TCZ	Elevation of TC, LDL-C, and triglyceride levels, no change in small dense LDL	(141)
2015	RA (n=21)	TCZ	Weight gain, alterated fat distribution, increased muscle mass	(135)
2018	RA (n=40)	TCZ	Comparable increases in different lipoproteins, no change in atherogenic index	(139)
2019	RA (n=50)	TCZ	Decrease in serum insulin levels and insulin/glucose ratio, improved insulin sensitivity	(134)
2020	RA (n=107)	TCZ	Increased total adiponectin and HMW adiponectin, increased body mass index and waist circumference but no parallel change in waist-to-hip ratio, no change in fat mass, increased muscle mass	(136)

RA, rheumatoid arthritis; TCZ, tocilizumab; MRA, a Humanized anti-IL-6 receptor antibody; HMW, high molecular weight; TC, total cholesterol; LDL-C, low-density lipoprotein cholesterol; T2DM, type 2 diabetes mellitus; HbA1c, glycated hemoglobin A1c.

During treatment with anti-IL-6 therapy, elevations in total cholesterol (TC), low-density lipoprotein cholesterol (LDL-C), and triglyceride levels have been observed during TCZ treatment for RA (138). This phenomenon has triggered a deeper investigation into the metabolic effects of TCZ. Although elevated lipid levels are usually considered indicators of increased risk for metabolic diseases, the situation seems to be different in the context of TCZ treatment. The raised lipid levels induced by TCZ do not seem to result in increased cardiometabolic risks, but instead may lower the risk of cardiovascular events. This paradox can be explained by multiple factors.

First, the anti-inflammatory effects of TCZ may partially mitigate the potential risks associated with elevated lipid levels. TCZ leads to changes in different lipoproteins with a comparable gain, thus not affecting the atherogenic index (139), and potentially even alleviating arterial stiffness in patients (140). Importantly, the key pathogenic factor of atherosclerosis (small dense LDL) is not influenced by TCZ (141). Also, the increase in the levels of high-molecular-weight (HMW) adiponectin during TCZ treatment serves as a positive indicator for the improvement in metabolic diseases (136), suggesting that TCZ may play an important role in metabolic regulation.

Furthermore, studies in obese patients and those intolerant to statins have shown that anti-inflammatory therapy with IL-6 inhibitors plays a positive role in improving metabolism and disease progression in these patients. In a cohort study involving 1,153 obese participants, serum lipoprotein(a) (Lp(a)), a severe independent risk factor for cardiovascular diseases, was significantly associated with IL-6 levels. After TCZ treatment, Lp(a) levels were considerably reduced in patients (10), a change attributed specifically to the inhibition of IL-6 rather than to the suppression of inflammatory immune responses. The latest follow-up study of 13,970 statin-intolerant high-risk cardiovascular patients demonstrates that inflammation has a greater predictive value than LDL-C for future cardiovascular events and mortality (142), highlighting the significance of anti-inflammatory therapies in ameliorating metabolic disorders and reducing cardiovascular risk.

In conclusion, the clinical trials discussed above suggest that the improvements in insulin sensitivity and inflammation levels resulting from IL-6 blockade may lead to clinical benefits that outweigh the risks associated with elevated lipid levels.

### 4.4 IL-6 regulates the RAAS

The overactivation of the RAAS leads to progressive renal damage. Angiotensin II (AngII) is the primary effector of RAAS, generated from angiotensin I under the action of angiotensin-converting enzyme. Studies have shown that IL-6 is necessary for the renal damage caused by aberrant activation of RAAS.

IL-6 plays a key role in AngII-induced renal damage and fibrosis. Studies have shown that AngII stimulates IL-6 secretion from renal tubular cells (97) and elevates IL-6 expression in rat kidneys (143). In the absence of IL-6, AngII was unable to induce phosphorylation of JAK2/STAT3 in RTECs (144) and in murine kidneys (145). And IL-6 blockade inhibited mesangial cell proliferation induced by AngII (146). In IL-6 knockout mice, AngII-induced hypertension and urinary albumin excretion were lessened (145, 147), accompanied by a notable reduction in vascular endothelial inflammatory injury and oxidative stress (148). Moreover, L-6 deficiency decreased AngII-induced expression of renal fibrotic genes in mice (34, 149), thereby mitigating chronic kidney lesions. Multiple studies have shown that IL-6 further amplifies intrarenal AngII action by elevating the expression of renal angiotensinogen (AGT) (144, 150, 151), creating a vicious cycle that ultimately results in progressive renal function decline.

The mechanism by which IL-6 mediates the effects of AngII has attracted considerable interest. Multiple studies confirm the critical role of IL-6-mediated JAK2/STAT3 signaling in AngII-induced kidney injury (144, 145, 152). Furthermore, JAK-STAT signaling also underlies the increase in AGT levels induced by IL-6 (144, 153). JAK2/STAT3 activation in the kidney has been identified as a facilitator of AngII-induced kidney injury in diabetic patients (154). Thus, it is possible that the IL-6/JAK/STAT signaling pathway is a key downstream signal in RAAS-induced kidney injury.

### 4.5 IL-6 regulates renal iron homeostasis

Ferroptosis, a form of iron-dependent cell death characterized by lipid peroxidation, has been increasingly recognized for its close association with the onset and progression of DKD (155). Ferroportin (FPN) and hepcidin play key roles in mammalian iron homeostasis. The activation of the IL-6/Ca axis has been shown to enhance the presence of FPN on the plasma membrane of human embryonic kidney cells, which promotes iron efflux, and removes Fpn from the membrane via upregulation of hepcidin to restore iron homeostasis (156).

IL-6 is recognized as an inducer of ferroptosis involved in intervertebral disc degeneration and mastitis diseases (157, 158). Epitope-mimicking peptides are believed to be effective in mimicking antibody binding sites, inducing active immunity (159). In kidney diseases, the specific epitope mimics of TCZ were found to increase ferritin level, decrease lipid oxidation levels and renal cell apoptosis, attenuating kidney fibrosis (73). Besides, IL-6 mediates signaling crosstalk between ferroptotic kidney cells and surrounding fibroblasts, accelerating renal fibrosis (160). In autoimmune kidney diseases, IL-6 helps immune cells evade ferroptosis, promoting excessive activation of immune cells and worsening kidney disease. Blockade of IL-6 promotes ferroptosis in B cells and shows therapeutic effects in lupus nephritis (161). These findings highlight the intricate involvement of IL-6 in the regulation of ferroptosis and suggest that the impacts of IL-6 on ferroptosis are context-dependent.

## 5 The current status of IL-6 inhibitors in clinical trials related to DKD

The intrinsic relationship between IL-6 and DKD has been substantiated through various aspects in the aforementioned laboratory studies. In the process of translating targeted IL-6 therapy for DKD into clinical application, relevant clinical trials are being gradually conducted. Current IL-6 inhibitors in clinical trials for kidney disease mainly consist of TCZ, clazakizumab (an anti-IL-6R antibody), ziltivekimab (an IL-6 ligand inhibitor), and baricitinib (a JAK inhibitor). As shown in **Table 3**, the majority of studies evaluate the safety and efficacy of anti-IL-6 therapy in pre- and post-kidney transplant patients (NCT01594424, NCT03444103, NCT00106639, NCT00658359, NCT04561986, NCT03859388, NCT04779957, NCT03867617, NCT03380962, NCT03380377). According to the available data, IL-6 inhibitors effectively reduce immune rejection in post-kidney transplant patients with good tolerability (162–166).

**Table 3:** IL-6 inhibitors in DKD-related clinical trials.

Interventions	Patients studied	Status/major outcomes	Enrollment	Phases	Trial registration	Study completion date
Tocilizumab	HS patients awaiting RT	Reduced antibody-mediated immune response, improved transplant success rate	10	1/2	NCT01594424	2015-05
	Patients with obesity and T2DM	Reversible reduction of meal- and exercise-induced GLP-1	56	Not applicable	NCT01073826	2016-06
	KTR with inflammation	Increase in Treg frequency, decrease in T-effector cell cytokine responses	33	2	NCT02108600	2018-12-16
	KTR	Active, not recruiting	12	1/2	NCT03867617	2029-06
	KTR	Recruiting	50	3	NCT04561986	2027-12
	KTR	Active, not recruiting	10	Not applicable	NCT03859388	2024-12-31
	Patients before or after graft nephrectomy	Recruiting	18	2	NCT04779957	2024-10
Clazakizumab	ESRD patients with diabetes or atherosclerosis	Reduction in inflammation markers	2310	2/3	NCT05485961	2028-12
	Patients with ABMR after RT	Completed, but results not disclosed	10	1/2	NCT03380377	2024-04-16
	KTRwith late ABMR	Decrease in donor-specific antibodies, improved eGFR decline	20	2	NCT03444103	2020-06-30
	KTR	Unmet Expectations	194	3	NCT03744910	2024-04-08
	HS Patients Awaiting RT	Active, not recruiting	20	1/2	NCT03380962	2025-07-30
Ziltivekimab	ESRD patients	Improvement in multiple inflammatory markers and thrombotic biomarkers	264	2	NCT03926117	2020-06-26
	people with CVD, CKD and inflammation	Recruiting	6200	3	NCT05021835	2026-01-29
	Chinese people with kidney disease and inflammation	Active, not recruiting	24	1	NCT05379829	2024-05-27
Baricitinib	DKD patients	Reduction in proteinuria levels, Alleviated renal inflammation	130	2	NCT01683409	2014-11
	CKD patients with hypertension	Recruiting	75	2	NCT05237388	2026-03-31
	DKD patients with severe albuminuria	Recruiting	20	2	NCT05897372	2025-06-01
	lupus nephritis	Recruiting	80	2/3	NCT05686746	2023-08-01
	lupus nephritis	Active, not recruiting	60	3	NCT05432531	2023-04-01
Tofacitinib	Patients after RT	Inhibition of acute rejection	61	2	NCT00106639	2006-07
	5 year of follow-up on post-RT patients	Long-term renoprotective effects	178	2	NCT00658359	2015-06

KTR, kidney transplant recipient; ABMR, antibody-mediated rejection; RT, renal transplantation; HS, highly-HLA sensitized; CVD, cardiovascular disease; DKD, diabetic kidney disease; CKD, chronic kidney disease; ESKD, end-stage kidney disease; T2DM, type 2 diabetes mellitus; GLP-1, glucagon-like peptide-1; eGFR, estimated glomerular filtration rate.

In addition, anti-IL-6 therapy also effectively improved inflammatory markers and proteinuria levels in patients with kidney disease, leading to a reduction of kidney inflammation. Previous results from a phase 2b clinical trial indicate that clazakizumab reduces serum inflammatory markers related to cardiovascular events in participants with ESKD complicated by diabetes or atherosclerosis (NCT05485961) (167). A Phase 3 trial is currently ongoing.

Furthermore, ziltivekimab shows improvements in multiple inflammatory markers and thrombotic biomarkers in ESKD patients with high cardiovascular risk (NCT03926117) (168). Trials are currently recruiting to assess the impact of ziltivekimab on cardiovascular risk in patients with cardiovascular disease, chronic kidney disease, and systemic inflammation (NCT05021835). The anti-inflammatory effects of TCZ have been evaluated in patients with post-kidney transplant inflammatory responses (164) (NCT02108600). Baricitinib has shown efficacy in reducing proteinuria levels and alleviating renal inflammation in T2DM patients with kidney diseases (169) (NCT01683409). Furthermore, studies are planned to evaluate the effect of baricitinib on urine protein in CKD (NCT05237388) patients and its impact on hard kidney endpoints in DKD patients with severe albuminuria (NCT05897372). Several studies (NCT05686746, NCT05432531) are planned to evaluate the effectiveness of baricitinib for lupus nephritis.

Overall, the results of these clinical trials provide robust evidence supporting the strategy of targeting IL-6 for treating DKD.

## 6 Conclusion

The current indications for IL-6 inhibitors have expanded from the initial rheumatoid arthritis, to more recent giant cell arteritis, and systemic sclerosis-associated interstitial lung disease (170, 171). Targeting IL-6 has shown significant potential for the development of treatments for immune-mediated inflammatory diseases. With the new concept of immunometabolism and the growing understanding of the crosstalk among immunity, iron metabolism and hemodynamics in preclinical studies, immunotherapy is highly likely to be a new breakthrough in treating DKD. The latest clinical trials are assessing the efficacy of IL-6 inhibitors on inflammatory markers relevant to cardiovascular and renal diseases.

In the investigation of targeted therapy strategies with anti-IL-6 agents for DKD, there are two points of concern. Firstly, further investigation is needed into the molecular mechanisms underlying the functions of the classical and trans-IL-6 signaling pathways. Both pathways transmit signals through the shared mediator gp130, but they can elicit distinct or even opposing effects. Thus, additional research at the molecular levels is necessary to elucidate the key mechanisms of this precise control and differential action, which would significantly advance the development of precision medicine for DKD. Some recent studies have focused on this. For example, Xu et al. have revealed the crosstalk between energy metabolism and IL-6 signaling, elucidating the molecular mechanisms underlying the distinct actions associated with reprogramming of glucose metabolism (172). Another study has demonstrated that changes in the binding parameters of the IL-6-gp130 receptor complex can lead to biased signaling (173). Specifically, modified cytokine-receptor binding dynamics can attenuate the pleiotropic effects of IL-6, thus mitigating potential side effects associated with IL-6-targeted treatments.

Secondly, in the development of IL-6-targeted therapy for DKD treatment, how to modulate the balance between the classical and trans-signaling pathways of IL-6 is also a challenging task. IL-6 trans-signaling-specific inhibitor olamkicept has been developed for treating ulcerative colitis with promising results in phase II clinical trials (174). An increasing number of studies are being redirected to the development of drugs that selectively inhibit IL-6 trans-signaling without affecting classical signaling. However, due to the diverse pathological manifestations in different diseases, targeting only the IL-6 trans-signaling pathway in DKD may not be the most effective approach, as demonstrated by the inefficacy of sgp130Fc in ameliorating insulin resistance induced by a high-fat diet in mice (131). In contrast, the recombinant IC7Fc, which is constructed with consideration of tuning of the balance between the two primary IL-6 pathways, has exhibited multiple beneficial effects in the context of type 2 diabetes. Therefore, in comparison to global inhibition of IL-6 or simply inhibition of the IL-6 trans-pathway, developing drugs that modulate the balance between IL-6 classical and trans-signaling pathways may be a more rational therapeutic strategy for DKD.
